# Composite Nanocellulose Fibers-Based Hydrogels Loading Clindamycin HCl with Ca^2+^ and Citric Acid as Crosslinking Agents for Pharmaceutical Applications

**DOI:** 10.3390/polym13244423

**Published:** 2021-12-16

**Authors:** Pichapar O-chongpian, Mingkwan Na Takuathung, Chuda Chittasupho, Warintorn Ruksiriwanich, Tanpong Chaiwarit, Phornsawat Baipaywad, Pensak Jantrawut

**Affiliations:** 1 Department of Pharmaceutical Sciences, Faculty of Pharmacy, Chiang Mai University, Chiang Mai 50200, Thailand; pichaparo@gmail.com (P.O.-c.); chuda.c@cmu.ac.th (C.C.); yammy109@gmail.com (W.R.); tanpong.c@gmail.com (T.C.); 2 Department of Pharmacology, Faculty of Medicine, Chiang Mai University, Chiang Mai 50200, Thailand; mingkwan.n@cmu.ac.th; 3 Cluster of Research and Development of Pharmaceutical and Natural Products Innovation for Human or Animal, Chiang Mai University, Chiang Mai 50200, Thailand; 4 Biomedical Engineering Institute, Chiang Mai University, Chiang Mai 50200, Thailand; phornsawat.b@cmu.ac.th

**Keywords:** nanocellulose fiber, hydrogel, low methoxyl pectin, sodium alginate, clindamycin

## Abstract

Biocomposite hydrogels based on nanocellulose fibers (CNFs), low methoxy pectin (LMP), and sodium alginate (SA) were fabricated via the chemical crosslinking technique. The selected CNFs-based hydrogels were loaded with clindamycin hydrochloride (CM), an effective antibiotic as a model drug. The properties of the selected CNFs-based hydrogels loaded CM were characterized. The results showed that CNFs-based hydrogels composed of CNFs/LMP/SA at 1:1:1 and 2:0.5:0.5 mass ratios exhibited high drug content, suitable gel content, and high maximum swelling degree. In vitro assessment of cell viability revealed that the CM-incorporated composite CNFs-based hydrogels using calcium ion and citric acid as crosslinking agents exhibited high cytocompatibility with human keratinocytes cells. In vitro drug release experiment showed the prolonged release of CM and the hydrogel which has a greater CNFs portion (C_2_P_0.5_A_0.5_/Ca + Ci/CM) demonstrated lower drug release than the hydrogel having a lesser CNFs portion (C_1_P_1_A_1_/Ca + Ci/CM). The proportion of hydrophilic materials which were low methoxy pectin and sodium alginate in the matrix system influences drug release. In conclusion, biocomposite CNFs-based hydrogels composed of CNFs/LMP/SA at 1:1:1 and 2:0.5:0.5 mass ratios, loading CM with calcium ion and citric acid as crosslinking agents were successfully developed for the first time, suggesting their potential for pharmaceutical applications, such as a drug delivery system for healing infected wounds.

## 1. Introduction

Cellulose is the most abundant polymer produced by plants and microorganisms [1]. Normally, cellulose is fibrous with intermittent crystalline and amorphous sections. The separation of fibers results in nanoscale cellulose substances known as nanocellulose, which exists nanocrystals (CNCs) and cellulose nanofibers (CNFs) [2]. Nanocellulose has high number of hydroxyl groups, high mechanical strength, renewability, and low cost [3]. For these reasons, nanocellulose has been considered an ideal nanostructure for making new high-value materials in many fields and gained much attention and interest from researchers. Nanocellulose can be used alone or combined with other polymers or materials for various fields, such as wound dressing, food, cosmetics, tissue engineering, energy, electrospinning, bioprinting, and so on [1,4,5]. This study focuses on CNFs, which are micrometer-long entangled fibrils that contain both amorphous and crystalline cellulose domains. More specifically, CNFs have many unique properties, such as biodegradability, biocompatibility, high strength and modulus mechanical properties, large specific surface area, ability to form a strong entangled nanoporous network, as well as swelling in water and water absorptivity [6]. Due to their attractive properties, CNFs were selected to use as a biopolymer and investigated for their applications to be used as a nanostructure polymer to form a hydrogel.

Hydrogel fabrication from CNFs alone is an ongoing challenge due to the lack of common solvents for their dissolution. However, nanocellulose can disperse in some strong polar solvents (especially water) due to the strong interaction between the surface hydroxyls and solvent molecules. However, the hydrogen bonding between nanofibers still leads to aggregation at the micro-level [7,8]. So far, achieving a good dispersion of CNFs in aqueous media is still a major challenge in developing CNFs-based hydrogel. The addition of polyethylene glycol (PEG) in CNFs could improve the dispersity of CNFs in the aqueous phase. PEG could physically adsorb onto the surface of nanocelluloses via hydrogen bonding [9].

Furthermore, CNFs-based hydrogels are formed by mixing with other polymers to form new nanocomposite materials. Liu et al. fabricated CNF derived from the TEMPO-oxidised method in conjunction with different types of hemicellulose galactoglucomannan, xyloglucan, and xylan crosslinkers to produce a series of nanocellulose hydrogels [10]. Similarly, Yang et al. produced CNF-polyacrylamide composites hydrogels by forming ionic and covalent bonds with multivalent cations [11]. Orasugh et al. have prepared hydroxypropylmethyl cellulose-based nanocomposites with cellulose nanofibrils as a packaging and transdermal drug delivery system [12]. Carlström et al. have blended and crosslinked gelatin with CNFs to produce scaffolds with tuned degradation rates and enhanced mechanical properties [13]. Thus, our main goal of this study was to seek an optimal formulation of CNFs-based hydrogel for pharmaceutical applications, which could be served as a material analogue to use as a carrier for drugs or other biomolecules. In this report, we have focused on the development of hydrogels by using three biopolymers, including CNFs, low methoxy pectin (LMP), and sodium alginate (SA), for fabricating the hydrogels with three crosslinking solutions: citric acid or CaCl_2_ or citric acid with CaCl_2_. We hypothesized that the combination of these biopolymers and CNFs-based hydrogels’ properties might lead to the invention of hydrogels with potential use as a drug carrier. After preparation, hydrogels were morphologically characterized, investigated for their gel content, swelling ratio, and mechanical strength, and tested for cytotoxicity in a human keratinocytes cell line. Then, clindamycin hydrochloride (CM), an active antibiotic against aerobic Gram-positive and anaerobic bacteria, mycoplasmas, and some protozoa [14], was used as a model drug and loaded into the selected CNFs-based hydrogel formulations. Assays of drug content and release profiles from these CM-loaded hydrogels were then performed.

## 2. Materials and Methods

### 2.1. Materials

Cellulose nanofibers (CNFs, white dry powder with nominal fiber width of 50 nm) were purchased from CelluloseLab, New Brunswick, Canada. Low methoxy pectin (LMP, degree of esterification = 29%) was purchased from Cargill^TM^, Saint Germain, France. Sodium alginate (SA, white granule) was purchased from Qindao Bright Moon Seaweed Group Co., Ltd., Qingdao, China. Calcium chloride (CaCl_2_) and citric acid monohydrate were purchased from RCI Labscan Ltd., Bangkok, Thailand. Distilled water was used as the solvent for preparing the hydrogels. All the other reagents were of analytical grade.

### 2.2. Preparation of CNFs-Based Hydrogels

CNFs-based hydrogels were prepared by varying the mass ratio between CNF and two polymeric precursors, LMP and SA. The compositions of the CNFs-based hydrogels are summarized in Table 1. Briefly, LMP and SA were dissolved in deionized water (DI) and heated to 60 ± 0.5 °C, hold for 1 h, and cooled to 35 ± 0.5 °C. The CNF powders were dispersed in 40% *w*/*w* PEG 1500 and mixed with the LMP and SA solution after which they were homogenized by a homogenizer (IKA T25 Ultra-Trurrax, IKA laboratory technology, Staufen, Germany) at 500 W. Homogeneous polymeric dispersions were obtained under homogenization at room temperature after 2 h. Each ten grams of a formulation were weighed and poured in 3 Petri dishes, envisioning three different crosslinking methods. The crosslinking solution, containing 0.5 M citric acid (Ci) or 3% *w*/*w* CaCl_2_ (Ca) or 0.5 M citric acid, and 3% *w*/*w* CaCl_2_ (Ci + Ca), was poured into each formulation. After 2 h, crosslinked hydrogels were taken out, and additionally washed with DI. Then, the excess surface DI was removed by gently blotting with a filter paper before being freeze-dried (Christ Beta 2-8 LD-plus, Osterode am Harz, Germany) for 24 h to obtain the crosslinked CNFs-based sponges. The CNFs-based hydrogels that demonstrated suitable absorption and tensile properties as well as low toxicity on human keratinocytes cell line were selected to load clindamycin hydrochloride (CM) as a model drug and investigated for drug content and in vitro drug release profile. Briefly, CM was simultaneously incorporated into the CNF dispersion to achieve a 1% *w*/*w* CM concentration then followed the steps described above.

### 2.3. CNFs-Based Hydrogels Characterizations

#### 2.3.1. Morphological Characterizations

After crosslinking, the digital images of the CNFs-based hydrogels were taken using a digital camera (Canon EOS 750D with an 18–55 mm lens, Canon, Inc., Tokyo, Japan). Scanning electron microscopy (SEM) images of coated CNFs-based hydrogels were acquired using JEOL JCM-7000 NeoScope™ Benchtop SEM (JEOL, Tokyo, Japan). Prior to imaging, uncoated hydrogels were mounted on aluminium stubs using double-sided carbon tape (NEM tape, Nisshin Co., Ltd., Tokyo, Japan) and coated with gold for 2 min then positioned on the stage in the imaging compartment of the device. Then, SEM images of all CNFs-based hydrogels were collected using a secondary electron detector at an acceleration voltage of 15 kV under low vacuum mode. Subsequently, a cross-section of CNFs-based hydrogels’ morphology was conducted at magnifications of ×100.

#### 2.3.2. CNFs-Based Hydrogels Thickness

The average thickness of hydrated CNFs-based hydrogels was determined using an outside micrometer (3203-25A, Insize Co, Ltd., Suzhou, China). Five random measurements were taken on each hydrogel. The average of the five values and their standard deviation (S.D.) of individual hydrogel was calculated. Thickness measurements were performed in triplicate.

#### 2.3.3. Mechanical Strength Test of CNFs-Based Hydrogels

The mechanical strength of hydrated CNFs-based hydrogels was tested using a texture analyzer, TX. TA plus (Stable Micro Systems, Surrey, UK). An individual sample holder was constructed to facilitate the measurements of 2 cm × 2 cm hydrogel samples. The hydrogel was fixed on a plate with a cylindrical hole with a 9.0 mm diameter (the area of the sample holder hole was 63.56 mm^2^). A cylindrical stainless probe (2 mm in diameter) with a plane flat-faced surface was used (with a probe contact area of 3.14 mm^2^). The texture analyzer was adjusted for the probe’s forward movement at a velocity of 1.0 mm/s. Measurement started when the probe had contacted the sample surface (triggering force). The probe moved on at a constant speed until the hydrogel was broken. The breakage of hydrogels was detected when the peak force of the texture profile analysis curve dropped. The applied force and the slope of the force–time curve were recorded. All the experiments were conducted at room temperature conditions (25 °C, 70% relative humidity). Five replicates were conducted for each hydrogel. The mechanical strength of the hydrogel was characterized by the puncture strength and Young’s modulus [15].

#### 2.3.4. Gel Content

The crosslinking efficiency was evaluated by gel content analysis in deionized water (DI) and phosphate buffer saline (PBS) pH 7.4. Freeze-dried CNFs-based hydrogels were cut into 2 cm × 2 cm dimension, and the initial dried weight (W_i_) was measured. Then, the hydrogels were immersed in the testing media at room temperature for 48 h and dried for 48 h in a 40 °C oven (Memmert GmbH Co., KG, Schwabach, Germany) until they reached a constant weight (W_f_). Gel content (GC) was calculated using the following Equation (1) [16]. The experiment was performed in triplicate under the same conditions and the average GC values were calculated.

GC (%) = W_f_/W_i_ × 100 (1)

#### 2.3.5. Swelling Ratio

The swelling ratio of freeze-dried CNFs-based hydrogels was determined by using a gravimetric method [17]. Initially, freeze-dried CNFs-based hydrogels were cut into 2 cm × 2 cm pieces, and their dry weights (W_d_) were measured. Each sample was immersed in a vial containing 20 mL deionized water (DI) and phosphate buffer saline (PBS) pH 7.4 at room temperature for 24 h. At certain intervals, the swollen hydrogels were withdrawn from DI or PBS. The wet weight of the swollen hydrogels (W_s_) was measured after the removal of excess surface DI or PBS by gently blotting with a filter paper. The maximum swelling degree (%) was defined by the following Equation (2). These tests were carried out in triplicate under the same conditions, and the average values were reported.

Maximum swelling degree (%MSD) = (W_s_ − W_d_)/W_d_ × 100. (2)

### 2.4. Cell Culture

An immortalized human epidermal keratinocyte cell line, HaCaT cell, was obtained from Cell Lines Service GmbH (Eppelheim, Baden-Württemberg, Germany). The cells were cultured in Dulbecco’s modified Eagle’s media (DMEM) (Thermo Fisher Scientific, Waltham, MA, USA), supplemented with 10 % fetal bovine serum (Merck KGaA, Darmstadt, Germany) and antibiotics (100 U/mL penicillin and 100 μg/mL streptomycin) (Thermo Fisher Scientific, Waltham, MA, USA), and incubated under a humidified atmosphere of 37 °C, 5% CO_2_.

### 2.5. Cell Viability Assay

Effects of the CNFs-based hydrogels on the viability of HaCaT cells were performed by using 3-(4,5-dimethylthiazol-2-yl)-2,5-diphenylte-trazolium bromide (MTT) (Sigma-Aldrich, Saint Louis, MO, USA). HaCaT cells were seeded in 96-well plates at a density of 2 × 105 cells per well and incubated for 24 h in complete DMEM. The indirect contact assay or elution test was performed by using the hydrogels extractable from the cell culture medium. The hydrogels were cut into a piece of 5 mm × 5 mm × 1 mm and sterilized by UV-irradiation for 30 min. Then, the hydrogel’s extract was prepared by soaking the hydrogel in DMEM medium for 48 h and subsequently filtered through a 0.22-µm syringe filter [18]. After that, HaCaT cells were treated with 200 µL CNFs-based hydrogel extract for 24 h. The cell viability was performed by MTT assay for assessing cell metabolic activity. Briefly, 200 μL of MTT reagent (0.4 mg/mL) dissolved in culture medium was added into each conditioned well for 1–2 h. Then, the culture supernatants were discarded, and 100 μL of 100% dimethyl sulfoxide (DMSO) was added to solubilize the formazan complex. The plate was then measured for absorbance at 570 nm using a microplate reader (BioTek Instruments, Winooski, VT, USA).

The CNFs-based hydrogel formulations that provided suitable gel content and maximum swelling degree with low cytotoxicity on HaCaT cells were selected to incorporate clindamycin (CM). Then, drug content, gel content, maximum swelling degree, cytotoxicity on keratinocyte cell line, and in vitro drug release of CNFs-based hydrogels containing CM were investigated.

### 2.6. CM Loading Content

Three randomly taken freeze-dried CNFs-based hydrogels containing CM (about 0.1 g of hydrogel) were added into vials containing 20 mL of PBS buffer solution (pH 7.4) and set aside until the hydrogels dissolved completely by using a magnetic stirrer (50 rpm) at room temperature for 48 h. The solutions were filtered through a 0.45-µm membrane filter and then diluted. The average amount of drug-loaded into hydrogels was analyzed using a UV-spectrophotometer (UV2600i, Shimadzu Corporation, Kyoto, Japan) at 202 nm. The CM contents in hydrogel formulations were determined from the standard curve prepared with CM solution in a concentration range of 0.025–0.1 mg/mL with a high linear regression (*r*^2^ = 0.9962). Each hydrogel sample was tested in triplicate. The CM content of the selected CNFs-based hydrogels was calculated with the following Equation (3):

(3) $$\mathrm{Drug}\;\mathrm{loading}\;\mathrm{content}\;\left(\%\right)=\frac{Amount\;of\;drug\;in\;hydrogel}{Theorectical\;drug\;content}\times 100$$

### 2.7. In Vitro Drug Release Profile and Release Kinetic

CNF-based Hydrogels containing CM in a square shape (1 cm × 1 cm) were immersed in 20 mL PBS buffer pH 7.4 at 37 ± 0.5 °C. The medium was stirred continuously with a magnetic bar at 50 rpm. The dissolution media (3 mL) was taken at predetermined times (0.5, 1, 2, 3, 6, 12, 24, 30, 36, 48, and 72 h), and the phosphate buffer saline (3 mL) was replaced. The samples were analyzed for CM release using a UV-Vis spectrophotometer (UV2600i, Shimadzu, Kyoto, Japan) at 202 nm wavelength. All dissolution experiments were performed in triplicate. The amount of released drug was calculated with the following Equation (4):

(4) $$\mathrm{Amount}\;\mathrm{of}\;\mathrm{released}\;\mathrm{drug}\;\left(\%\right)=\frac{Amount\;of\;released\;drug\;at\;the\;specific\;time}{Amount\;of\;drug\;in\;hydrogel}\times 100\;$$

Drug release kinetics and mechanism were described by different drug release kinetic models. The first 60% of the data of the drug release was fixed with zero-order, first-order, Higuchi and Korsmeyer–Peppas models described in Equations (5)–(8), respectively [19].

The zero-order model describes that the dissolution rate is constant over the period of time and independent from drug concentration. The equation for the zero-model release is shown in Equation (5):

*Q_t_* = *Q*_0_ + *k*_0_*t* (5)

where: *Q_t_* is the cumulative amount of drug release at each predetermined time

*Q*_0_ is the initial amount of drug

*k*_0_ is zero-order kinetic constant

*t* is time

In first-order release kinetic, the drug release rate typically depends on its concentration. The first-order release kinetic was shown in Equation (6):

(6) $$\log Q_{0}-\log Q_{t}=\frac{k_{1}t}{2.303}$$

where: *Q_t_* is the cumulative amount of drug release at each predetermined time

*Q*_0_ is the initial amount of drug

*k*_1_ is first-order kinetic constant

*t* is time

The Higuchi model is developed to study the drug release of water-soluble and slightly water-soluble drugs incorporated in solid and/or semi-solid matrixes. The equation for the Higuchi model is shown in Equation (7):

(7) $$Q_{t}=k_{H}t^{\frac{1}{2}}$$

where: *Q_t_* is the cumulative amount of drug release at each predetermined time

*k*_H_ is Higuchi kinetic constant

*t* is time

The Korsmeyer–Peppas model relates to the exponential drug release and the fractional drug release. It is used to explain the drug release from a polymer matrix. The Korsmeyer–Peppas model is shown Equation (8):

(8) $$\frac{Q_{t}}{Q_{0}}=kt^{n}$$

where: *Q_t_/Q*_0_ is the fraction of drug release

*k* is structural and geometrical constant

*t* is time

### 2.8. Statistical Analysis

The represented data are expressed as mean ± standard deviations (S.D.). The one-way ANOVA test was carried out using SPSS^®^ statistics software version 17.0 (IBM Corporation, Armonk, NY, USA) to analyze the statistical significance of the results. The *p* level less than 0.01 were considered statistically different.

## 3. Results

### 3.1. Preparation and Morphological Characteristics of CNFs-Based Hydrogels

**Figure 1:**
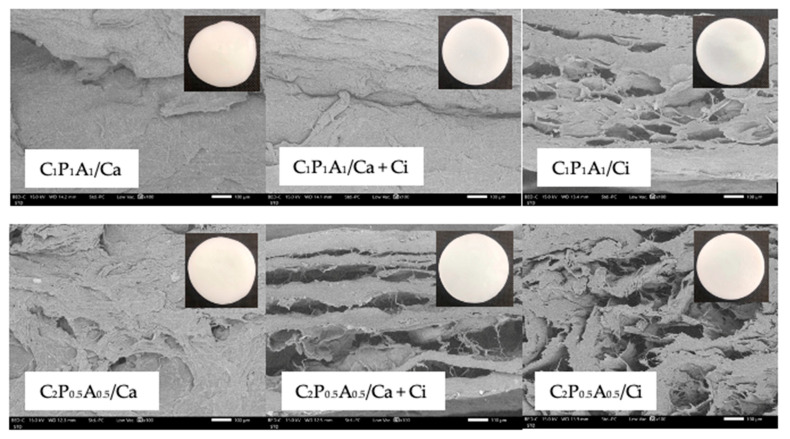
Influence of the composition and crosslinking agent of the hydrated CNFs-based hydrogels and the freeze-dried CNFs-based hydrogels composed of C:P:A with mass ratio 1:1:1 and 2:0.5:0.5 by SEM micrographs (cross-sections) at 100×.

In the present study, we developed CNFs-based hydrogels as a material-analogue to use as a carrier for drugs or other therapeutic biomolecules. Hydrogel-forming biopolymers, including CNFs, LMP, and SA were used, and twelve different hydrogel formulations were prepared by chemically crosslinked using 3% *w*/*w* CaCl_2_ solution (Ca), 3% *w*/*w* CaCl_2_ in 0.5 M citric acid solution (Ca + Ci) or 0.5 M citric acid solution (Ci). The addition of PEG also made CNFs easily dispersed and helped to increase hydrogel’s flexibility [9]. Therefore, following our optimization of CNFs-based hydrogel formulations using the appropriate ratio of each polymer and technique, flexible and non-brittle crosslinked hydrogels were successfully prepared. As shown in Figure 1, all formulations of C_1_P_1_A_1_ and C_2_P_0.5_A_0.5_ CNFs-based hydrogels were homogeneous and translucent. Meanwhile, the formulations with composition of CNFs less than 1 mass ratio, which was C_0.5_P_2_A_0.5_ and C_0.5_P_0.5_A_2_ formulations exhibited rough surface and an irregular shape which may be due to incomplete hydrogel formation (Figure S1). Moreover, a large standard deviation (>2 mm) of the thickness of C_0.5_P_2_A_0.5_ and C_0.5_P_0.5_A_2_ formulations (Table S1) indicates that these formulations were not suitable for further mechanical testing. This study found that the mass ratio of CNFs is a key parameter that influences the structure of the composite hydrogels that contained LMP and SA. Thus, C_1_P_1_A0_1_ and C_2_P_0.5_A_0.5_ CNFs-based hydrogels were focused. The SEM micrographs of C_1_P_1_A0_1_ and C_2_P_0.5_A_0.5_ CNFs-based hydrogels revealed the polymer matrix morphology and porosity with architectures strongly dependent on the composition of the hydrogels on the crosslinking method, as visible in Figure 1. CNFs appeared to be well-integrated with LMP and SA since no separation phase was noticed. The addition of crosslinking agents such as Ca, Ca + Ci, or Ci solutions was an important parameter that strongly influenced the morphology and porosity [17]. The morphological evaluation of the CaCl_2_ crosslinked hydrogels revealed a significant difference in the morphology of the polymeric structuring when comparison with citric acid crosslinked samples. CaCl_2_ crosslinked hydrogels revealed less porosity and dense polymer microstructures. For micrographs of citric acid crosslinked hydrogels, they revealed a highly porous morphology. In this study, we mixed 3% *w*/*w* CaCl_2_ in 0.5M citric acid solution as crosslinking agents. We found that the morphology of the CNFs-based hydrogels crosslinked with Ca + Ci showed higher and homogeneous internal porosity than Ca crosslinked hydrogels.

### 3.2. Mechanical Properties of CNFs-Based Hydrogels

To investigate the influence of biopolymers ratio and type of crosslinking agent on the mechanical properties of CNFs-based hydrogels, puncture strength and Young’s modulus were evaluated (Table 2). Compared with the same crosslinking agent, the puncture strength of CNFs-based hydrogels decreased with increasing CNFs portion. Despite the crosslinking method, LMP- and SA-richer hydrogels exhibited a higher puncture strength. This may be due to C_1_P_1_A_1_ hydrogels having more crosslinked junctions within the polymeric matrix than C_2_P_0.5_A_0.5_ hydrogels. Moreover, CNFs-based hydrogels using Ci as a crosslinking agent (C_1_P_1_A_1_/Ci and C_2_P_0.5_A_0.5_/Ci) exhibited lower puncture strength than other CNFs-based hydrogels using Ca and Ca + Ci as crosslinking agents. The decrease of the puncture strength was believed to be due to decreased crosslink density [20]. From Young’s modulus values, it can be seen that the most elastic CNFs-based hydrogels were C_2_P_0.5_A_0.5_/Ci. It can be observed that the citric acid-crosslinked hydrogels present higher elasticity compared to Ca and Ca + Ci-crosslinked hydrogels. However, Young’s modulus values between CNFs-based hydrogels using Ca and Ca + Ci have no statistically significant difference. Our results suggested that the type of crosslinking agent and the ratio between the matrix components have a strong influence over the material strength.

### 3.3. Gel Content and Swelling Properties of CNFs-Based Hydrogels

**Figure 2:**
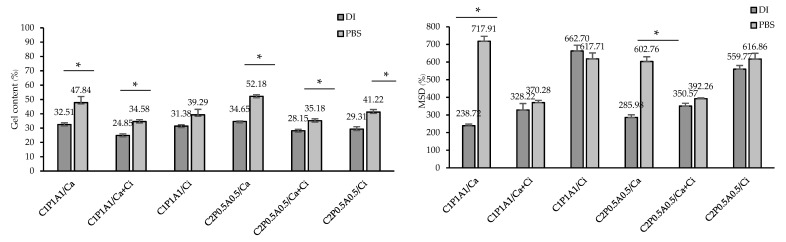
Gel content (**left**) and maximum swelling degree (MSD) (**right**) of CNFs-based hydrogels in DI and PBS at significant level of * *p* < 0.01.

The structural stability of CNFs-based hydrogels was assessed in terms of GC (%) in two different media: DI and PBS in both physiological pH of 7.4, and the results were displayed in Figure 2. According to the gel fraction analysis, the formulations using Ca as a crosslinking agent (C_1_P_1_A_1_/Ca and C_2_P_0.5_A_0.5_/Ca) exhibited higher %GC (~45% to 55%) than the formulations crosslinked by Ca + Ci and Ci both in DI and PBS. The higher structural stability might be explained by a dense polymer matrix and strong microstructure due to its ability to generate a crosslinked network by hydrogen bonds and ionic bonds. The formulations using Ca + Ci and Ci as crosslinking agents exhibited lower %GC (~20% to 40%) because of the highly porous network and hydrogen bonds interaction with immersion media. The number of hydrophilic groups (–OH or –COOH) of the CNFs, pectin, and sodium alginate crosslinked by Ca + Ci or Ci was abundant. In the formulations containing citric acid, more than one hydrogen bond are formed by interactions with many functional groups per structural unit, while Ca can interact with only one group from each structural unit. The increase of the hydrophilic groups resulted in the increase of polarity of the composite and the enhancing solubility in water, respectively. Hence, the gel content is lowered [17,21]. Our results also found that the different ratios among the three polymers had revealed slightly significant differences in gel content.

The swelling properties of freeze-dried CNFs-based hydrogels were evaluated after 24 h, and the maximum swelling degree (%MSD) in two different media, DI and PBS, is presented in Figure 2. In DI, the formulations using Ci as a crosslinking agent (C_1_P_1_A_1_/Ci and C_2_P_0.5_A_0.5_/Ci) exhibited higher %MSD (~560% to 660%) than the formulations crosslinked by Ca and Ca + Ci due to the highly porous network and hydrogen interaction with water. Generally, hydrogels with a low degree of crosslinking showed higher water uptake ability since the highly crosslinked structure couldn’t sustain much water within the gel structure [22,23]. In PBS, the %MSD of the hydrogels using Ci or Ca + Ci as a crosslinking agent revealed no substantial differences compared to the %MSD in DI. However, the formulations using Ca as a crosslinking agent (C_1_P_1_A_1_/Ca and C_2_P_0.5_A_0.5_/Ca) exhibited the lowest %MSD in DI (~235% to 285%) because of low hydrogen bonds in their network and dense polymer matrix reducing the water permeability. Nonetheless, the swelling capacity of the formulations using Ca as a crosslinking agent in PBS provided higher %MSD (~600% to 700%) because ion exchange promotes an additional relaxation of the network and enhances the swelling [17,24]. The formulations using Ca + Ci as crosslinking agents exhibited %MSD around 300% to 400% because they exhibited strong microstructure owing to generating a crosslinked network by hydrogen bonds and ionic bonds influencing swelling properties.

### 3.4. Effects of CNFs-Based Hydrogels on Cell Viability of HaCaT Cells

**Figure 3:**
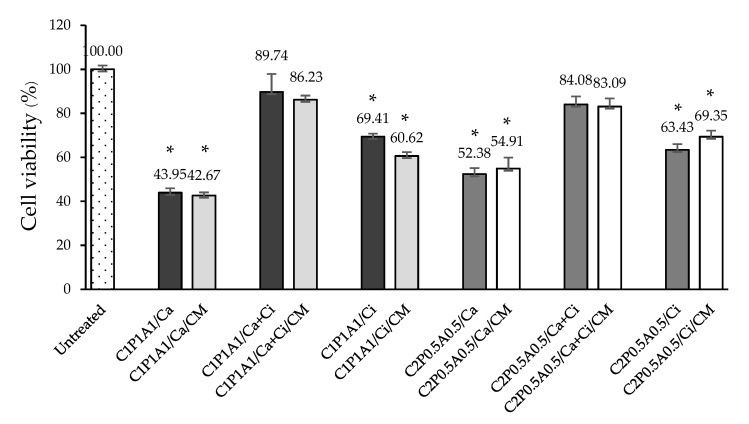
HaCaT cell viability of CNFs-based hydrogels and CNFs-based hydrogels containing CM at a significant level of * *p* < 0.01 in comparison with the untreated group.

Considering the novel composite nanocellulose fiber-based hydrogels and crosslinking strategy, an evaluation of cellular response to the hydrogels was fundamentally required. We performed an MTT assay to measure the cell viability of keratinocytes, using HaCaT cells to specifically select the CNFs-based hydrogels that are not toxic to the cells for further experiments. The mitochondrial dehydrogenase performance measurement of CNFs-based hydrogels-treated cells cultured in complete media was performed. For CNFs-based hydrogels without CM, the results revealed good biocompatibility of the assessed materials in contact with HaCaT cells indicating that the combination between CNFs and LMP and SA using Ca + Ci as crosslinking agents was favorable for cell viability more than 80% (Figure 3). The same statistical difference was observed for CNFs-based hydrogels containing CM using Ca + Ci as crosslinking agents in comparison with CNFs-based hydrogels without CM. However, significantly decreased cell viability for Ca or Ci crosslinking agent was observed in C_1_P_1_A_1_/Ca, C_2_P_0.5_A_0.5_/Ca, C_1_P_1_A_1_/Ci and C_2_P_0.5_A_0.5_/Ci with and without CM (~40% to 60% cell viability). This may be due to calcium and citric in the hydrogel samples inducing alkaline (pH ~ 8) and acidic (pH ~ 4) media, respectively, which leads to diminishing cell viability and cell number. Meanwhile, the media pH of CNFs-based hydrogels using Ca + Ci (C_1_P_1_A_1_/Ca + Ci and C_2_P_0.5_A_0.5_/Ca + Ci) were around 6–7. Lönnqvist et al. studied the effect of alkaline and acidic pH on keratinocyte viability. Similar to our study, they showed that alkaline or acidic conditions are detrimental to cells even with a limited duration of exposure [25].

**Figure 4:**
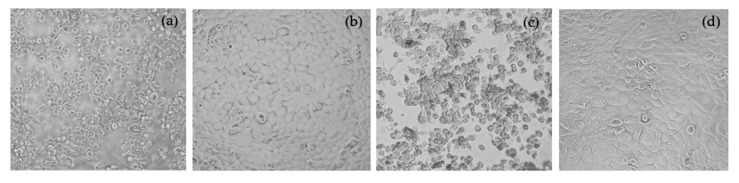
Morphology of HaCaT cells treated with CNFs-based hydrogels containing CM using Ca (**a**), Ca + Ci (**b**), Ci (**c**) as crosslinking agents and untreated (**d**) groups.

Interestingly, the cell viability results were in accordance with the action of CNF-based hydrogels on HaCaT morphology as examined by phase-contrast microscopy. The morphology of HaCaT cells treated with CNFs-based hydrogels containing CM using Ca + Ci was similar to the untreated group, suggesting that these hydrogels showed a non-toxic effect to the cells (Figure 4b). In contrast, morphological changes of HaCaT cells treated with CNFs-based hydrogels using Ca or Ci were observed in a larger proportion of cells swelling and round-out, indicating necrosis. Some revealed shrinkage of cells with unclear nuclei, suggesting apoptosis (Figure 4a,c). Our results also found that the ratio among the three polymers had a minor effect on cell viability. The cells treated with DMSO as vehicle control (untreated group) at consistent concentrations existing in the CNFs-based hydrogels group did not cause any difference in cell viability. Thus, C_1_P_1_A_1_/Ca, C_2_P_0.5_A_0.5_/Ca, C_1_P_1_A_1_/Ci and C_2_P_0.5_A_0.5_/Ci hydrogels were not selected for our further study.

### 3.5. Drug Content and CNFs-Based Hydrogels Containing CM Characteristics

**Figure 5:**
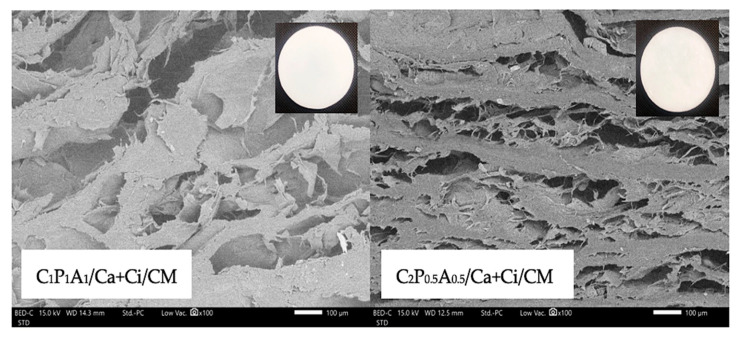
Gross appearance and SEM images of cross-section at 100× of C_1_P_1_A_1_/Ca + Ci/CM (**left**) and C_2_P_0.5_A_0.5_/Ca + Ci/CM (**right**).

The clindamycin HCl (CM) contents in the selected CNFs-based hydrogels were determined and shown in Table 3. C_1_P_1_A_1_/Ca + Ci/CM and C_2_P_0.5_A_0.5_/Ca + Ci/CM hydrogels demonstrated more than 80% drug content, with C_2_P_0.5_A_0.5_/Ca + Ci/CM exhibited significantly higher drug contents than C_1_P_1_A_1_/Ca + Ci/CM. This may be due to the greater porosity in the C_2_P_0.5_A_0.5_/Ca + Ci/CM structure than C_1_P_1_A_1_/Ca + Ci/CM hydrogel (Figure 5). The high porosity in the hydrogel structure is linked to a higher drug loading in the hydrogel matrix [26]. The percent gel content of both CNFs-based hydrogels containing CM was similar in DI and PBS with a slightly different in comparison with the C_1_P_1_A_1_/Ca + Ci and C_2_P_0.5_A_0.5_/Ca + Ci hydrogels without CM. However, %MSD in DI and PBS of C_1_P_1_A_1_/Ca + Ci/CM were significantly higher than C_2_P_0.5_A_0.5_/Ca + Ci/CM. This may be due to the higher proportion of alginate and pectin. The hydrogen bonds formed by the interactions between alginate and pectin and water molecules could entrap media into the intramolecular space of the hydrogel structure. The maximum swollen occurs when the intracellular space is filled with media [27,28]. The gross appearance and SEM images of the selected formulation, C_1_P_1_A_1_/Ca + Ci/CM and C_2_P_0.5_A_0.5_/Ca + Ci/CM, are shown in Figure 5. After the crosslinking procedure, C_1_P_1_A_1_/Ca + Ci/CM and C_2_P_0.5_A_0.5_/Ca + Ci/CM demonstrated homogeneity and translucency with smooth surfaces hydrogels. The cross-sectional SEM images of the freeze-dried C_1_P_1_A_1_/Ca + Ci/CM and C_2_P_0.5_A_0.5_/Ca + Ci/CM exhibited an entangled nanoporous network in the hydrogels’ structure. The crystal structure of clindamycin HCl was not observed in the matrix of either hydrogel.

### 3.6. CM Release Profile and Kinetic

**Figure 6:**
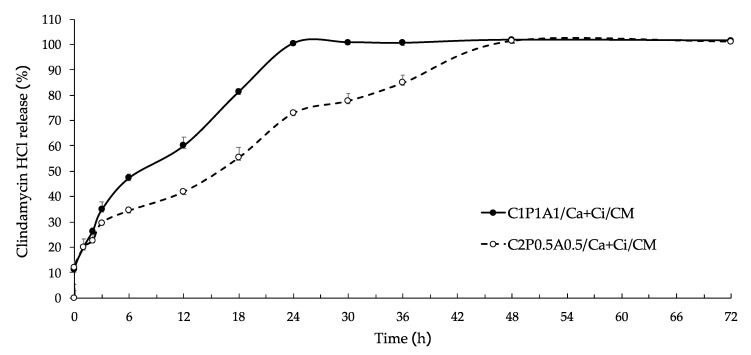
Clindamycin hydrochloride release profile of C_1_P_1_A_1_/Ca + Ci/CM and C_2_P_0.5_A_0.5_/Ca + Ci/CM hydrogels.

The drug release of profiles of C_1_P_1_A_1_/Ca + Ci and C_2_P_0.5_A_0.5_/Ca + Ci hydrogels containing CM are shown in Figure 6. Both formulations exhibited a similar pattern. The drug release indicated that both C_1_P_1_A_1_/Ca + Ci and C_2_P_0.5_A_0.5_/Ca + Ci hydrogels showed prolonged drug release. Overall, the C_1_P_1_A_1_/Ca + Ci and C_2_P_0.5_A_0.5_/Ca + Ci gradually released CM in 3 h and then slightly released to about 100%. From 3 to 48 h, the C_1_P_1_A_1_/Ca + Ci significantly released CM more than the C_2_P_0.5_A_0.5_/Ca + Ci. For example, at 6 h, the C_1_P_1_A_1_/Ca + Ci released CM of about 47%, whereas C_2_P_0.5_A_0.5_/Ca + Ci displayed 34%. However, the C_2_P_0.5_A_0.5_/Ca + Ci released CM around 100% within 48 h. The slower drug release of the C_2_P_0.5_A_0.5_/Ca + Ci hydrogel formulation could be explained by polymer compositions. Both formulations were comprised of pectin and alginate, which are hydrophilic polymers. On the other hand, they also contained nanocellulose fiber, which is not soluble in water. The proportion of hydrophilic material in the matrix systems influences on drug release [29]. In another study, researchers have investigated tramadol release from hydroxypropyl methylcellulose (HPMC) matrices with and without ethyl cellulose (water-insoluble polymer). They concluded that the HPMC matrices with ethyl cellulose displayed slower drug release due to the presence of hydrophobic polymer reduced permeation of the solvent molecules and decreased diffusion of the drug from polymeric matrices, respectively [30]. Possibly, the hydrogel composition containing higher ratio of CNFs may slow down the dissolution of CM. Consequently, the C_1_P_1_A_1_/Ca + Ci exhibited significantly faster CM release than that of C_2_P_0.5_A_0.5_/Ca + Ci.

The release behavior of CM in this study was investigated by various models, for instance, zero-order, first-order, Higuchi, and Korsmeyer–Peppas models. The release rate constant and correlation coefficient (R^2^) values are shown in Table 4. According to the models, the most appropriate model to evaluate CM release from the CNFs-based hydrogels was the Higuchi model due to higher R^2^. In this study, the Higuchi model suggested that the CM release mechanism was a diffusion from the polymeric matrix [31]. Furthermore, the C_1_P_1_A_1_/Ca + Ci showed a higher release rate constant (K) than the C_2_P_0.5_A_0.5_/Ca + Ci. This corresponds to the drug release profile that displayed higher CM release from C_1_P_1_A_1_/Ca + Ci than C_2_P_0.5_A_0.5_/Ca + Ci hydrogel. The data were also fixed with the Korsmeyer–Peppas model to explain the possible release mechanism with n value. Theoretically, *n* ≤ 0.45 corresponds to a Fickian diffusion mechanism, 0.45 < *n* < 0.89 to a non-Fickian transport, *n* = 0.89 to Case II (relaxational) transport, and *n* > 0.89 to super case II transport. In this study, the n values of C_1_P_1_A_1_/Ca + Ci and C_2_P_0.5_A_0.5_/Ca + Ci were 0.8020 and 0.8466, respectively, which corresponded to non-Fickian transport referring that the solute transport process in which the polymer relaxation time is approximate the characteristic solvent diffusion time. This means that the solvent absorption and active compound release depend on polymer swelling and polymer/solvent couple viscoelastic properties (Mario and Gabriele, 2005) [32].

## 4. Conclusions

CNFs-based composite hydrogels were successfully developed with low methoxyl pectin and sodium alginate using calcium and citric acid as crosslinking agents. Among 12 hydrogel formulations, C_1_P_1_A_1_/Ca + Ci and C_2_P_0.5_A_0.5_/Ca + Ci demonstrated suitable gel content and maximum swelling degree with low cytotoxicity on HaCaT cells, and were selected to load CM and evaluate for drug content and in vitro drug release profile. C_1_P_1_A_1_/Ca + Ci/CM and C_2_P_0.5_A_0.5_/Ca + Ci/CM showed CM contents greater than 80%. The in vitro drug release data revealed that the cumulative drug release percentage decreased with the increase of CNFs portions in the composite CNFs-based hydrogels with C_2_P_0.5_A_0.5_/Ca + Ci/CM as the prolonged CM release profile up to 3 days. Based on the present study, we concluded that CNFs/LMP/SA using Ca + Ci composite hydrogels can be used for pharmaceutical applications as a prolonged release system in transdermal drug delivery. However, the developed CNFs-based hydrogels also require further studies to evaluate their stability at different pH (acidic, neutral and basic), in vitro antibacterial activity, and/or in vivo treatment of skin infection.

## Figures and Tables

**Table 1:** Composition of different CNFs-based hydrogels.

Mass Ratio	Polymer Composition/40 g			Sample Code		
	CNFs	LMP	SA	Calcium Crosslinking	Calcium and Citric Acid Crosslinking	Citric Acid Crosslinking
1:1:1	0.4	0.4	0.4	C_1_P_1_A_1_/Ca	C_1_P_1_A_1_/Ca + Ci	C_1_P_1_A_1_/Ci
2:0.5:0.5	0.8	0.2	0.2	C_2_P_0.5_A_0.5_/Ca	C_2_P_0.5_A_0.5_/Ca + Ci	C_2_P_0.5_A_0.5_/Ci
0.5:2:0.5	0.2	0.8	0.2	C _0.5_ P_2_A_0.5_/Ca	C _0.5_ P_2_A_0.5_/Ca + Ci	C _0.5_ P_2_A_0.5_/Ci
0.5:0.5:2	0.2	0.2	0.8	C_0.5_P_0.5_ A_2_/Ca	C_0.5_P_0.5_ A_2_/Ca + Ci	C_0.5_P_0.5_ A_2_/Ci

Note: CNFs = cellulose nanaofibers, LMP = low methoxyl pectin and SA = sodium algonate.

**Table 2:** Thickness, tensile strength, and Young’s modulus of CNFs-based hydrogel formulations.

Formulations	Thickness (mm)	Puncture Strength (N/mm^2^)	Young’s Modulus (N/mm^2^)
C_1_P_1_A_1_/Ca	6.896 ± 0.520 ^a^	0.827 ± 0.058 ^a^	0.159 ± 0.004 ^a^
C_1_P_1_A_1_/Ca + Ci	3.370 ± 0.187 ^b^	0.602 ± 0.034 ^b^	0.110 ± 0.007 ^a^
C_1_P_1_A_1_/Ci	3.464 ± 0.324 ^b^	0.115 ± 0.006 ^c^	0.053 ± 0.002 ^b^
C_2_P_0.5_A_0.5_/Ca	5.109± 0.382 ^a^	0.384 ± 0.008 ^d^	0.068 ± 0.014 ^b^
C_2_P_0.5_A_0.5_/Ca + Ci	3.470 ± 0.109 ^b^	0.228 ± 0.008 ^e^	0.066 ± 0.002 ^b^
C_2_P_0.5_A_0.5_/Ci	3.376 ± 0.075 ^b^	0.063 ± 0.003 ^f^	0.038 ± 0.002 ^c^

For each test, average values with the same letter are not significantly different. Thus, average values with the different letters, e.g., ^‘a’^ or ^‘b’^ or ^‘c’^ or ^‘d’^ or ^‘e’^ or ^‘f’^ are statistically different (*p* < 0.01).

**Table 3:** Drug content, gel content and maximum swelling degree (MSD) of CNFs-based hydrogels containing CM.

Formulations	Drug Content (%)	Gel Content (%)		MSD (%)	
		DI	PBS	DI	PBS
C_1_P_1_A_1_/Ca + Ci/CM	83.21 ± 2.42 ^a^	24.94 ± 8.31 ^a^	23.71 ± 2.02 ^a^	522.22 ± 188.80 ^a^	472.51 ± 30.98 ^a^
C_2_P_0.5_A_0.5_/Ca + Ci/CM	94.21 ± 4.05 ^b^	21.54 ± 0.43 ^a^	25.50 ± 0.79 ^a^	345.04 ± 55.74 ^b^	350.18 ± 18.48 ^b^

For each test, average values with the same letter are not significantly different. Thus, average values with the different letter, e.g., ^‘a’^ or ^‘b’^ are statistically different (*p* < 0.01).

**Table 4:** Release kinetic data of the CNFs-based hydrogel containing CM in various drug release models.

Kinetic Models	Parameters	Samples	
		C_1_P_1_A_1_/Ca + Ci/CM	C_2_P_0.5_A_0.5_/Ca + Ci/CM
Zero-oder	R^2^	0.9004	0.9247
	*k*_0_ (h^−1^)	17.1810	17.8590
First order	R^2^	0.7244	0.7673
	*k*_1_ (h^−1^)	7.0787	13.6510
Higuchi	R^2^	0.9960	0.9839
	*k*_H_ (h^1/2^)	18.3460	13.550
Korsemeyer-Peppas	R^2^	0.8981	0.9423
	*k*(h^−*n*^)	0.0598	0.0361
	*n*	0.8020	0.8466

## Data Availability

The study did not report any data.

## Supplementary Materials

The following are available online at https://www.mdpi.com/article/10.3390/polym13244423/s1, Figure S1: Gross appearance and SEM images of the cross-section at 100× of CNFs-based hydrogels that composed of C:P:A with mass ratio 0.5:2:0.5 and 0.5:0.5:2, Table S1: Thickness of CNFs-based hydrogels that composed of C:P:A with mass ratio 0.5:2:0.5 and 0.5:0.5:2 formulations.
