# Supplementary material for: Composite Nanocellulose Fibers-Based Hydrogels Loading Clindamycin HCl with Ca2+ and Citric Acid as Crosslinking Agents for Pharmaceutical Applications
Source: Polymers (Basel). 2021 Dec 16;13(24):4423. doi: 10.3390/polym13244423 (PMC8707948; doi:10.3390/polym13244423)
Supplement: Supplementary file 1 [file polymers-13-04423-s001.zip › polymers-1486594-supplementary.pdf]

polymers

MDPI

Supplementary Materials: Composite Nanocellulose Fiber-Based Hydrogels Loading Clindamycin HCl with Ca^{2+} and Citric Acid as Crosslinking Agents for Pharmaceutical Applications

Pichapar O-chongpian [1], Mingkwan Na Takuathung [2], Chuda Chittasupho [1,3], Warintorn Ruksiriwanich [1,3], Tanpong Chaiwarit [1], Phornsawat Baipaywad [4] and Pensak Jantrawut [1,3,*]

1 Department of Pharmaceutical Sciences, Faculty of Pharmacy, Chiang Mai University, Chiang Mai 50200, Thailand; pichaparo@gmail.com (P.O.-c.); chuda.c@cmu.ac.th (C.C.); yammy109@gmail.com (W.R.); tanpong.c@gmail.com (T.C.)
2 Department of Pharmacology, Faculty of Medicine, Chiang Mai University, Chiang Mai 50200, Thailand; mingkwan.n@cmu.ac.th
3 Cluster of Research and Development of Pharmaceutical and Natural Products Innovation for Human or Animal, Chiang Mai University, Chiang Mai 50200, Thailand
4 Biomedical Engineering Institute, Chiang Mai University, Chiang Mai 50200, Thailand; phornsawat.b@cmu.ac.th
* Correspondence: pensak.amuamu@gmail.com or pensak.j@cmu.ac.th; Tel.: +66-53944309

Citation: O-chongpian, P.; Na Takuathung, M.; Chittasupho, C.; Ruksiriwanich, W.; Chaiwarit, T.; Baipaywad, P.; Jantrawut, P. Composite Nanocellulose Fibers-Based Hydrogels Loading Clindamycin HCl with Ca^{2+} and Citric Acid as Crosslinking Agents for Pharmaceutical Applications. *Polymers* **2021**, *13*, 4423. https://doi.org/10.3390/polym13244423

Academic Editor: Luis García-Fernández

Received: 15 November 2021
Accepted: 14 December 2021
Published: 16 December 2021

Publisher's Note: MDPI stays neutral with regard to jurisdictional claims in published maps and institutional affiliations.

Copyright: © 2021 by the authors. Licensee MDPI, Basel, Switzerland. This article is an open access article distributed under the terms and conditions of the Creative Commons Attribution (CC BY) license (https://creativecommons.org/licenses/by/4.0/).

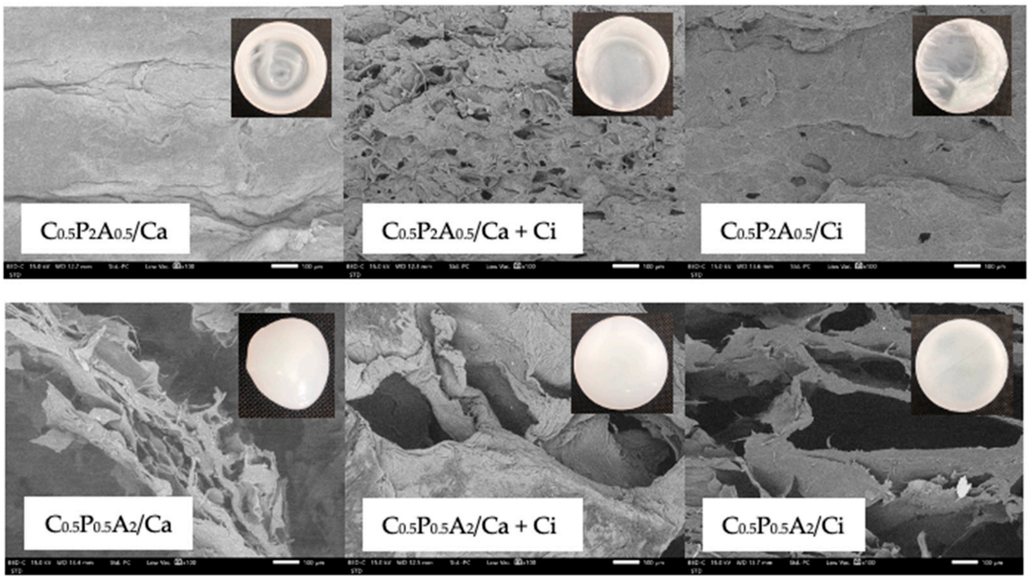

Figure S1. Gross appearance and SEM images of cross-section at 100× of CNFs-based hydrogels that composed of C:P:A with mass ratio 0.5:2:0.5 and 0.5:0.5:2.

Table S1. Thickness of CNFs-based hydrogels that composed of C:P:A with mass ratio 0.5:2:0.5 and 0.5:0.5:2 formulations.

**Formulations**	**Thickness (mm)**	**Puncture strength)N/mm²(**	**Young's modulus (N/mm²)**
$C_{0.5}P_2A_{0.5}$/Ca	3.990 ± 2.199	NA	NA
$C_{0.5}P_2A_{0.5}$/Ca+Ci	3.742 ± 2.151	NA	NA
$C_{0.5}P_2A_{0.5}$/Ci	3.541± 2.146	NA	NA
$C_{0.5}P_{0.5}A_2$/Ca	11.740 ± 5.453	NA	NA
$C_{0.5}P_{0.5}A_2$/Ca+Ci	5.558 ± 2.453	NA	NA
$C_{0.5}P_{0.5}A_2$/Ci	3.916 ± 2.674	NA	NA

NA (Not applicable): A large standard deviation of the thickness of CNFs-based hydrogels that composed of C:P:A with mass ratio 0.5:2:0.5 and 0.5:0.5:2 indicates that these formulations were not suitable for machanical testing.
